# Using Video-Reflexive Ethnography to Engage Hospital Staff to Improve Dementia Care

**DOI:** 10.1177/2333393618785095

**Published:** 2018-07-11

**Authors:** Lillian Hung, Alison Phinney, Habib Chaudhury, Paddy Rodney

**Affiliations:** 1 Simon Fraser University, Vancouver, British Columbia, Canada; 2 University of British Columbia, Vancouver, British Columbia, Canada

**Keywords:** dementia, hospitals, reflexivity, teamwork, video

## Abstract

In this article, we discuss how video-reflexive ethnography may be useful in engaging staff to improve dementia care in a hospital medical unit. Seven patients with dementia were involved in the production of patient-story videos, and fifty members of staff (nurses, physicians, and allied health practitioners) participated in video-reflexive groups. We identified five substantial themes to describe how video-reflexive groups might contribute to enacting person-centered care for improving dementia care: (a) seeing through patients’ eyes, (b) seeing normal strange and surprised, (c) seeing inside and between, (d) seeing with others inspires actions, and (e) seeing with the team builds a culture of learning. Our findings suggest that video reflexivity is not only useful for staff engagement but also effective in enhancing team capacity to enact person-centered care in the hospital setting.

## Introduction

Recent reports in the media and research literature have repeatedly shown evidence of poor care being provided to older people with dementia in acute care hospitals (e.g., Dewing & Dijk, 2016; Francis, 2013). Consequently, critics have questioned the ability of hospital staff to deliver person-centered care for patients with dementia (Francis, 2013; Venturato, Moyle, & Steel, 2011). Research points to the need to enable engagement of frontline staff and create supportive organizational contexts to achieve and sustain person-centered care (Dewing & McCormack, 2017; Kim & Park, 2017).

Person-centered care involves acknowledging and honoring the personhood of individuals by using a holistic approach to meet their needs (Dewing & McCormack, 2017). In person-centered care, paying attention to the social environment to meet psychosocial needs is considered as important as accomplishing medical tasks to optimize the patient’s well-being (Kitwood, 1997). The VIPS model (acronym as very important persons) has been used in staff education to support practice development in this area (Brooker, 2006). It highlights the intrinsic value of human life, framing care as an individualized approach to understanding the patient’s perspective and drawing on principles of positive social psychology to improve well-being. Although this focus on the person has been an important conceptual shift over the past 20 years, McCormack and McCance (2017) correctly pointed out that contextual factors, such as organizational culture and the learning environment, pose great challenges for staff in the provision of person-centered care. Because the clinical care for patients in acute care hospitals involves complex processes and is highly contextual, the application of person-centered care theory requires involving staff to motivate them to engage in ongoing adaptation to meet emergent and unpredictable challenges (McCormack & McCance, 2017).

Bushe and Marshak (2018) supported the view held by McCormack and McCance (2017) and noted that helping clinical teams increase their adaptive capacity to deal with complex and uncertain changes is an essential part of organizational development in health care. Adaptive capacity refers to making adjustments in practice to suit emerging circumstances (Iedema, Mesman, & Carroll, 2013). Research suggests that bringing people together to take a step back, reflect, and propose local solutions to confront challenges is a way to develop adaptive capacity (Hung, Lee, Au-Yeung, Kucherova, & Harrigan, 2016). Iedema (2011) underscored that our capacity to act is enhanced by our power to be affected. Put differently, social conditions for learning that support sharing in each other’s activities and experiences are important for enhancing team capacity.

Moreover, the importance of patients’ perspectives has been increasingly emphasized in research. Studies have shown that patients contribute to research quality and care improvement because they offer useful insights and unique perspectives that health care professionals might not otherwise have (Collier, Sorensen, & Iedema, 2016; Wyer et al., 2017). Hearing narratives or stories from patients can encourage critical reflection by staff and lead to reflexive learning in clinical practice (Adams, Robert, & Maben, 2015; Hung et al., 2017). Iedema (2011) explained that patients’ stories portray what clinicians do as clinicians’ actions are experienced—that is what lends power to patients’ stories. However, the voices of patients with dementia have often been ignored and overlooked in hospital care due to stigma and stereotyping (Dewing & McCormack, 2017; Gove et al., 2018). “Patients’ stories” told directly by patients with dementia are different from “stories of patients” told by clinicians. Excluding the direct perspectives of patients with dementia in research can reduce the relevance and impact of the research findings, as first-person accounts have the power to inspire, humanize, compel action, and challenge assumptions (Iedema et al., 2013). Based on the doctoral research of Hung (2017), in this article, we examine the use of video-reflexive ethnography as a way for staff to connect with and reflect on patients’ stories of care experiences and explain how this approach supported their engagement to improve dementia care in a medical unit.

### Video-Reflexive Ethnography (VRE)

VRE involves filming what happens in patient care and showing the footage to clinicians to stimulate discussion about the potential for improvement (Iedema et al., 2013). Iedema et al. (2015) described VRE as a methodology informed by the theory that practice change occurs when people are enabled to *question* and *disrupt* their habituated ways of being and acting. Video emphasizes the complexity of everyday care and allows the researcher to capture the situated nature of human experience and action in dynamic ways (Knoblauch, Tuma, & Schnettler, 2015). Unlike deficit models, “exnovation” in VRE allows a social process to drive change by making the strengths and potentials of the team visible to themselves (Iedema et al., 2013). In contrast to innovation, which relies on external resources, exnovation posits that clinicians themselves already have resources that only need to be revealed, “let out of the box”—not in-novated but ex-novated (Iedema et al., 2013).

As Pink (2013) pointed out, because video ethnography allows recording of subtle and simultaneously complex proceedings in time and space, it is increasingly used in projects to empower people to implement change. Iedema et al. (2013) described VRE as a practical science that makes “moment-to-moment complexity [in clinical care] visible, speakable, and (re)designable” (p. 13). In VRE, staff come together in video-reflexive groups to review videos filmed in situ and engage in team reflection, developing new and mutually agreeable ways (reflexivity) to care for patients. Iedema (2011) described reflection as thinking back to the event and addressing it, while reflexivity is “our capacity to monitor and affect events, conducts and contexts in situ” (p. 84). In contrast to reflection, reflexivity highlights adopting a socializing and contextualizing perspective on one’s actions (Iedema et al., 2013). Rather than being top-down, video-reflexive groups focus on the insight and experiential knowledge of frontline staff and patients from the bottom up (Wyer et al., 2015).

Carroll, Iedema, and Kerridge (2008) used VRE to study clinical communication on intensive care units (ICUs) with clinicians and nurse managers in Australia. Using a handheld digital camera, the researchers recorded eight 1-hour communication periods on four units. The researchers showed the video clips to staff in “reflexive sessions.” Professional communication and patient safety issues were identified, and the staff participants developed and implemented strategies to improve communication and other processes. The staff emphasized the positive impact of reflecting on the video recordings. As a result of the video feedback, the staff implemented changes in the patient handoff to improve communication and patient safety in transition.

More recently, Collier and Wyer (2016) used videos to involve patients in their patient safety research. Collier was concerned with end-of-life care, while Wyer focused on infection control. Collier filmed patients’ narratives of care. Through watching video footage with patients, Collier made decisions with patients about what would be shown to staff so that they could identify better ways to care for patients at the end of life. Wyer filmed patient and staff clinical interactions and showed the footage to patients who analyzed it for infection risks. Wyer fed back both the original footage and footage of the patients’ commentary on the event to the staff who cared for them. Staff were then able to see the benefits of patient involvement in infection control and to develop better ways to involve patients. In both these studies, patients identified safety risks that the clinicians were unaware of and shared strategies that contributed to the safety of their care. Thus, the use of videos in health research is not new. However, making videos for staff learning featuring the patient stories of patients with dementia admitted to hospitals is new.

### Purpose

Informed by the work of Collier and Wyer (2016), the study examined how VRE may support staff engagement to improve dementia care in a hospital unit. Hung worked under the supervision of three professors, the research committee, and coauthors of the article. Two techniques of VRE were used in the study: (a) filming go-along interviews conducted with patients with dementia and (b) conducting team discussion after watching the patient videos in reflexive groups. Specifically, this article offers a rich analysis of staff experiences in video-reflexive groups and of how VRE can contribute to creating team commitment and actions to enact person-centered care for improving dementia care.

### Research Design

The study was designed as appreciative action research underpinned by principles of positive collaboration and reflexivity (Reed, 2008). Appreciative action research is a type of action research that focuses on potentials, opportunities, and the strength of the participants for facilitating change. VRE was used to engage patients, staff, and leaders in co-inquiry; it offered a way to understand the complexity of dementia care through patients’ perspectives and allowed the team to explore possible solutions together in reflexive sessions that focused on exploring the strengths and potential within existing practice.

### Setting

The research was conducted in a large urban teaching hospital in Canada. The unit was a 31-bed medical ward that provides assessment and treatment to a general population of patients requiring medical and nursing care. Patients generally are admitted for symptoms of medical and/or mental health illnesses such as fractures, pneumonia, heart and lung diseases, dementia, delirium, and depression. The care team includes physicians, a patient-care coordinator, a nurse educator, nurses, care aides, an occupational therapist, a physiotherapist, and a social worker.

### Research Preparation and Recruitment

Hung, as a clinical nurse specialist in the hospital, had a good working relationship with the staff and patients on the unit, which contributed positively to recruitment. Two information sessions were held to provide details of the research and answer questions. The project had support from management and frontline leadership. Research posters were placed on the unit walls and information brochures were given to staff, patients, and families. Nurses who knew the patients well helped with purposive patient recruitment, identifying a diverse group of potential participants who had different subtypes and stages of dementia, various physical functional abilities, and different types of social backgrounds. Hung met with each patient participant, explained the purpose of the research and the processes that were involved, and answered questions. When patients agreed to take part, she then booked a time to conduct interviews and make videos with each person (*n* = 7).

As for staff recruitment, all staff were invited to participate in the research and were free to join one or more reflexive groups. Nine reflexive groups were undertaken, with most scheduled as two back-to-back sessions between 2:00 and 3:00 p.m. This meant that while some of the staff members attended the group, other team members on the unit could attend to the patients’ needs. Physicians participated in a separate group at lunch time, which was more convenient for their work schedule. Focus groups were conducted in the early and later phases of the research. In the early phase, focus groups were used to identify the dreams and hopes of the team. In the later phase, focus groups were also used to codevelop actions for change.

### Participants

The seven patient participants had different types of dementia and were at different stages of the disease. Three of the patients were men and the other four were women. Fifty members of staff, including nursing staff (30), physicians (15), and allied health workers (five) attended the video-reflexive groups. The physicians attended a separate group to accommodate their schedules.

### Generating the Videos

Each patient was interviewed with video recording 2 or 3 times, generating a total of 210 minutes of video footage. The interviews took place in public spaces such as the corridors and activity room in the medical ward. Hung used a handheld camera to film the environment, following the patient participant’s lead. She asked each patient what he or she liked and disliked about the hospital environment. The production of go-along videos (Hung, 2015) offered a participant-driven, multisensory approach to capture the complexities of the patients’ experiences as they were interacting with the hospital environment. The filming in go-along interviews was a collaborative coproduction with patients with dementia who took an active role to determine what would be captured in the filming and interview. In contrast to traditional interviews conducted away from the hospital environment, go-along interviews allowed the materials in the environment to serve as prompts for the patients with dementia who otherwise might have had difficulty remembering details to talk about. Moreover, Hung would occasionally ask prompting questions to allow the patients’ embodied actions to be clarified. For example, she saw that a patient flinched when a staff member walked past too quickly. She then asked the patient, “I saw you pulled back; tell me what happened.” Together, these strategies resulted in video recordings of patients’ richly detailed stories about how they experienced and interacted with the hospital environment in the medical unit (Hung, 2015).

The next step involved giving all patient participants the opportunity to view their own footage and gain their permission to use the footage for team learning. Three patient participants and one family member watched their videos, but the other patients and families did not wish to review the videos. The patients and family members who reviewed the videos offered no additional comments. All patients and their families gave permission for the use of footage for local reflexive sessions and broader academic and educational purposes.

Hung then categorized the acquired videos of the patients’ stories, edited them into clips, labeled them, and transcribed them in NVivo 11 software (QSR International). She subsequently cataloged each video clip in terms of a theme that represented a patient story. For example, one patient story was about how the clutter in the hospital hallways hinders patients’ function. Another video clip showed a patient’s story of being restrained in a wheelchair and his opinion about using restraints for fall prevention.

### Generating the Reflexive-Group Data

In the video-reflexive groups, video clips of 5 to 10 minutes were projected onto a screen to allow a group of six to 10 staff members to watch together. The purpose was to provide staff participants with an opportunity to discuss and reflect on what could be learned from the patients’ stories shown in the videos. After viewing the video clips, Hung facilitated a discussion, using open-ended questions and prompts. Staff were asked the following questions: What are your thoughts after hearing what this patient said about the care environment? What are your feelings and emotions? What could we learn from this? From your perspective, what should be done to make an improvement? These group discussions were audio recorded and transcribed verbatim.

### Analysis

Based on the transcripts and video data, Hung conducted coding, using both deductive and inductive methods. For the deductive coding, she used sensitizing concepts identified in the literature to identify patterns. At the same time, she added new codes inductively to signify specific narrative content. The analysis process involved an ongoing movement iteratively and systematically from parts to the whole, and from the whole to parts, to check for disparities and common patterns. She then chose exemplars to represent substantial themes. Hung held regular meetings with three academic supervisors to discuss data generation and the analysis.

We worked diligently to ensure that the analysis was systematic, and rigorous thinking was embedded in a full range of activities. To ensure credibility, Hung applied member checking, a technique that involves returning common themes and key results to participants to check for resonance with their experiences. Furthermore, Hung kept a research journal to record personal reflections. Biweekly research meetings helped us to keep up-to-date with the data analysis and to challenge individual assumptions. From the results of the analysis, Hung then worked with the staff in a series of focus groups to codevelop actions to improve care for patients with dementia.

## Ethical Considerations

This study received approval from the research ethics committees of the university and the local health authority. The use of the video method to conduct research with people with dementia involves multiple complex ethical matters, but the researchers agree with Swaffer (2014) that not including the direct perspective of people with dementia in research hinders the validity of the knowledge produced about them.

To address the challenge of filming people with dementia in research, we followed ethics guidelines from the literature on dementia research (Dewing, 2007) and visual ethics on filming people with dementia (Puurveen, Phinney, Cox, & Purvest, 2015). We obtained proxy consent from substitute decision makers for people who did not have capacity in legal terms to consent, and we gave people with dementia opportunities to decide their level and type of involvement in the research. Thus, the process of obtaining consent was an ongoing process that included the initial written consent and ongoing verbal assent processes prior to and during filming (Dewing, 2007). For example, Hung sought assent before each session to remind the patients with dementia about the purpose of the research and their right to withdraw at any time. She carefully monitored verbal and nonverbal cues about the acceptability of the research activities. As Puurveen et al. (2015) emphasized, both ongoing consent and observation skills are important in the use of the video method with people with dementia.

Because the go-along interviews were conducted in the public spaces of the unit (e.g., corridors), the identities of the patient participants were likely known to others. We explained this risk to the patients and gave them the option of using videotaping or not in the go-along interviews. No one declined videotaping; all patient participants expected the video data should be used to teach others about their experiences and inform change.

We made the intention to use the data, including video footage, for academic and education purposes clear in the consent and data release form and explained it to all participants. The patient participants and their families signed a separate data release form to give permission to use the video recordings for purposes of staff education and academic knowledge dissemination. Staff members who attended the video-reflexive groups signed written consent forms. We gave all participants the option to waive their confidentiality and to be identified to acknowledge their contribution. We have used the real names of those who signed the waiver and pseudonyms for those who chose to remain anonymous.

## Results

**Figure 1.**
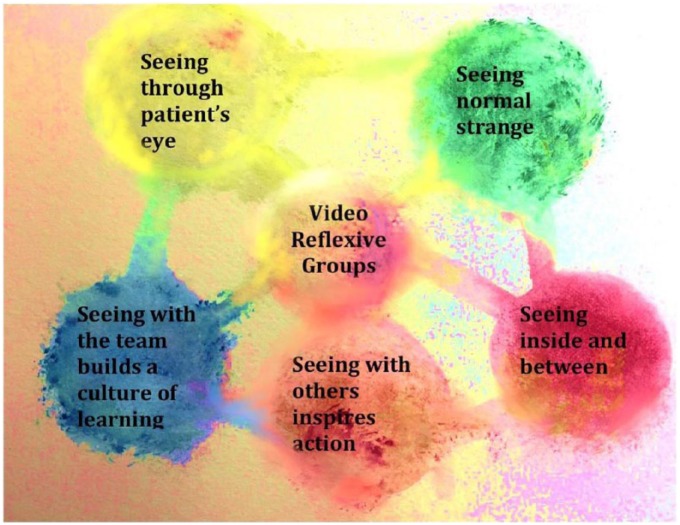
Seeing in 5 ways.

The aim of this article is to examine the role of VRE in creating commitment and actions to develop person-centered care in the medical unit. Our analysis indicated that five interrelated themes were important for team engagement in the development of person-centered care: (a) seeing through patients’ eyes, (b) seeing normal strange and surprised, (c) seeing inside and between, (d) seeing with others inspires actions, and (e) seeing team support builds a safe culture for learning (See Figure 1).

### Seeing Through Patients’ Eyes

Seeing through patients’ eyes refers to the empathetic meanings that emerged in the context of emotionally shared and embodied experiences. In the video-reflexive sessions, team members attended to how feelings were expressed by the verbal narratives and nonverbal embodied expressions. The video footage enabled patients to tell their stories in a visceral way. One nurse, Sharnjit, commented,

From the screen, it feels like we’re looking at the environment from the patient’s eyes, looking at the environment and walking in that video. It seems like he’s scared to go to the other end of the hallway because it’s dark. I think I see the problems, people rushing by, the sounds of people screaming or crying and the physicalness of it.

The staff were intrigued by how their professional perspective could be very different from that of the patients. A staff member, June, commented,

When you see it in the video you realize, wow, when we come to work, we see everything as useful tools for ourselves, and then when I see it from his eyes, it is not for him this place. The stuff is confusing and disabling. The hallways are so crowded, it’s really sterile and just kind of oppressive. The place is more tailored to us, not the patients.

The video recordings offered staff an immediate connection with the patients’ experiences and enabled staff to appreciate the difficulties that patients with dementia encountered. Examples of stigma, stereotyping, and misunderstanding were brought up for discussion: “I feel really bad for him. He wanted just to talk and not be judged. He looked intimidating; people misunderstood him. There’s a stigma attached to his appearance” (Mary, nurse). In the reflexive sessions, staff in different disciplines shared what they learned from hearing the narratives and viewing the movements of the scene as it unfolded from moment to moment. Their comments triggered deep empathetic reflections in the group. An occupational therapist, Carola, said,

What I noticed in the video is there was nowhere for him to go. Everything is not for him. The bed in the hallway wasn’t for him; the equipment’s not there for him. Nothing, there is no place to sit and relax. Now I understand where the aggression comes from, you know? I would get angry if I was tied down or I wasn’t allowed to do other things that other people can do. I can see he’s frustrated; it makes me feel sad for him.

Other staff spontaneously echoed the impact of the video. After a few minutes of watching the clips, they realized, “We can learn a lot from the patients themselves.” Another nurse, Bernard, added, “It made us realize the hospital environment can be very distracting and confusing to our patients.”

### Seeing Normal Strange and Surprised

After viewing the film clips, the staff remarked that they found new interpretations for the phenomenon that was taken for granted. The reflexive group sessions provided space and time to allow staff to generate new insights and give meaning to what otherwise might have gone unnoticed. Staff reported that the video recordings cast an unexpected and surprising light on things. Many of the staff said they had not seen the significance of the issues being faced by the patients until they saw the video clips in the group. A nurse, Maria, explained it in this way:

Looking at that video, I’m like, I can hear everything right now. Just watching it from here feels like my body was there. But when I’m actually in the hallways, I don’t seem to be bothered. Walking in the hallway, it feels normal to us. But, when we watch this video, I can see, oh my goodness, oh my gosh, there’s so many things, If I was him; I don’t know what to think. This opens my eyes. It’s good to reflect. We need to reflect more.

Another staff member, Ashley, said, “I had no idea of what it was like for him. He didn’t say escape, but, to me, I would want to escape. Wow! So, I am surprised with that kind of feeling.”

By staff watching and discussing the patients’ stories, and gaining a heightened sense of their experiential perspective, the videos drew attention to areas of presumption. A nurse, Glenda, illustrated this by saying, “Well, when I work I don’t really realize that’s how it feels. Even when people scream, we’re like, oh that’s just who they are. We got used to it and became too tolerant. It felt like normal.” A nursing aide, Nancy, added, “I didn’t realize the patients were lined up seating in front of the nursing station. It’s so hard to converse sideways, so uncomfortable; some can’t even turn their head.”

Team members reported that the dialogue in the reflexive group made them observe patients differently. One nurse coordinator, Roselin, remarked,

The conversations here made us see the patients not as dementia persons, but the person as a whole. And the part about the stigma and how we think and talk about people, we really have to be sensitive to that.

A nurse, Bernard, added,

For me what stood out is how we are caught up in the day-to-day task, like, how we do the handover or in our report, calling someone as aggressive. Now through our discussions, we are more aware of the bigger picture and we know more about the person.

One member of staff said, “I am so surprised to see the other side of Helen [patient].” Helen was labeled an “elopement risk and physically aggressive.” Helen told a volunteer that she wanted to go home. She did not understand why people held her down and gave her injections. In her care plan, she was to be given an injection when she approached the door. In the video, Helen was pleasant and social as she participated in an art workshop. She worked with a volunteer and painted a beautiful bird. A staff member commented, “Wow, she is actually teaching, contributing, and making the painting with the young volunteer together. This is amazing!” The video reflexivity changed how the team saw Helen. After that, Helen’s care plan was changed, and she received no more injections when approaching the door.

### Seeing Inside and Between

The reflexive groups served as a dialogic process that allowed for open exchanges of viewpoints and personal experiences. Staff said that they liked the reflexive sessions because they could hear other people’s opinions. Nurses stated that the discussions allowed them to think about and compare their care approaches with some of the problems. Furthermore, viewing the videos brought the staff to more affective dimensions of learning. A nurse, Gracita, stated,

It’s a bit emotional when you’re talking about these things. It’s quite private in some ways, your thoughts about these kinds of things, so when you can look into the thought process between people you work with, I think you feel more connected.

By comparing the exchanges of experience, the team learned much more about patients as persons. A few nurses spoke about how easy it is to let assumptions and misconceptions affect how individuals might be perceived and approached. People had different ideas and interpretations and used different approaches. The operationalization of person-centered care requires a form of skilled sense-making to understand the meaning behind behavioral symptoms. In the groups, the staff from different disciplines tried to combine what they knew individually with new ideas generated from the group. By watching the videos and discussing the patients’ stories, the staff gained a heightened sense of having things in common and a feeling of team commitment. The video aroused a shared awareness of the complexity of clinical situations in which they were all involved. They learned to appreciate the complexity of the clinical environment and the patients’ experiences:

When you know that we’re all working to a similar goal, you feel more comfortable also talking to people. If you feel like you know them a little bit better, it’s easier to ask them questions. You don’t feel kind of intimidated to ask anything. Then it’s better for everybody. Working as a team. I think it makes a huge difference. We know we’re there for each other. (Isaac, nurse)

### Seeing With Others Inspires Actions

The video-reflexive groups motivated affective learning and inspired actions in two ways. First, the groups allowed the staff to think, see, and feel together as a group. Second, affective learning was important to cause changes in the ways of being. Staff participants used emotive words such as feeling sad for the patient, feeling annoyed, and feeling frustrated. The staff reflected that specific aspects of the environment mattered. A nursing aide, Prem, commented, “We can’t just ignore what was going on in the environment. It has meanings for them. Now I feel I am more aware of the environmental factors. I am enlightened.”

Physicians spoke about what they heard and viewed in the videos that resonated with the everyday narratives of their patients. One physician, Maria, commented, “Every day one of my patients will say that they haven’t slept all night because of the noise and screaming or calling out. I think that people were coming in, we set them up for failure in that environment.”

It was evident that the intense emotions in the video-reflexive groups generated energy for everyone to make changes. During the discussions, many offered possible solutions. A nursing aide spoke about another hospital that organized their linens with shelves. Many spoke about the need for facilities and space for meaningful activities. Most agreed with the idea to expand volunteer services and provide dementia training for volunteers. A physician, Michael, suggested putting signage on the floor because “a lot of older patients . . . are kyphotic, and they’re looking at the ground, [and] it’s hard for them to see the number of the room that they’re at.”

The following statement by a nurse, Jane, illustrates how the subtle embodied expression sensitized staff’s perspective about a patient’s reality: “I see he flinched when someone all of [a] sudden shows up right at his face. That teaches me to slow down, not come up on somebody all of [a] sudden or don’t pass by them too fast.”

Seeing meant more than just looking. Video reflexivity acted as a springboard for learning and problem solving. A nurse, Cecilia, commented, “Now I learned that the environment, like how the noise can make patients feel, affecting their ability to think as well. It makes me pause to think again what causes my patient’s behaviors.” In response to that comment, another nurse, Roselin, said,

We should start thinking of if we could have little nooks that are more like people would have in home. We should have a cozy seat, or something to read, or something to do rather than stare at the hospital stuff, just the equipment we need.

Another nurse Brenda added, “Paintings and color would bring out the feelings of a more humanized place.” After the reflexive sessions, staff worked with Hung to develop design solutions and had funding approved by the hospital foundation to paint the walls and buy furniture to improve the physical environment of the medical unit.

### Seeing With Team Support Builds a Safe Culture for Learning

One important aspect of a safe culture for learning is trust. The group reflection gave the staff an opportunity to discuss successes and failures. The trust that was built between the team members, where individuals knew they were not alone and they had others on the team to support them, helped to build the culture of safe learning. None of the new knowledge would likely take root unless the culture and priorities of the hospital system were aligned. Inescapably, the adoption of new ways of working relies on a safe culture of learning. A few members of staff reported that they felt safer and more confident to question some aspects of the current state of matters and discuss their concerns with their colleagues. Others said that the reflexive groups helped them build a collective mindfulness for future practice. A unit clerk, Georgia commented,

We know that we have support and that the co-workers will help us out if something happens. So, we don’t put anyone at risk or get hurt. We just share our ideas, like, okay, what should we do in similar situations; we can give suggestions for possible solutions or try different approaches. We can help each other to keep everyone safe. I think that you can care for patients better when you feel connected to the people you’re working with.

The staff clearly explained that the reflexive groups gave the team an opportunity to build trust, which is essential for creating a climate of safety and openness. This team capacity is similar to what Mesman (2011) describes as exnovation, a way to promote safety by focusing on strengthening what is safe practice in the team. A nurse, Bernard, added,

It’s teamwork. With a team, you can kind of say we’re on the same page and we all work together and for the same goals. It’s kind of nice to see the problems in here as a team, work out solutions together. So, we feel connected. It’s like you know that when you do something new and creative, someone is there to support you. I think the positives come from everybody, and [the] manager. When we have the same attitude, we know we’re supported.

The affective component of the sense of team spirit made it easier for team members to ask questions and share their knowledge. Thus, this kind of untapped team intelligence can become unlocked.

## Discussion

In this article, we demonstrate the effectiveness of VRE in engaging staff in making collective commitments and taking action to improve dementia care by developing person-centered care. The findings show that the clinical team in the medical unit already had resources among themselves to provide person-centered care for their patients with dementia. Like any acute wards, the medical unit is highly structured, routinized, and fast-paced. Video reflexivity enabled the team to take a step back to pause and question what the hospital environment provided to patients with dementia. As Iedema (2009) suggested, VRE embeds a pragmatic pedagogic philosophy with a practitioner-driven and outcomes-based change strategy. Through five ways of seeing in team reflexivity, staff were motivated and engaged in developing person-centered care. Video of patient narratives acted not only as a light to illuminate the hidden issues but also as a catalyst to spark collective energy to find shared solutions to make improvements.

This study offers a new and unique contribution to the knowledge base of VRE and dementia care by using the first-person voices of patients with dementia (a rarely recruited and marginalized group) in VRE for team learning and practice development. Similar to the suggestion of Wyer et al. (2017), showing patients’ comments to staff can enable the team to consider how they might tackle complex situations in new ways. Viewing the videos of patients’ stories opened a space and encouraged the staff to talk about their practice and the actions of others. Learning was apparent in the reflexive groups, as the staff participants were affected by the footage they watched. Staff learned to appreciate and consider the perspectives of patients, heightening their empathy and desire for action to care for patients. In a recent study by Scerri, Innes, and Scerri (2016), the authors used appreciative inquiry to implement person-centered care in two hospital wards. Instead of using patient stories told by patients firsthand, they asked the staff and families to discuss the care being provided to persons with dementia in the hospital. Although we agree with the findings of Scerri et al. (2016) that learning from people’s experiences can be helpful for change, we believe that the effect of showing footage of patients’ interactions with the hospital environment and hearing the experiences described directly by patients is even more powerful.

The video of patient stories encouraged the staff participants’ critical reflection, allowing them to consider their experiences in ways that are not possible with traditional didactic education. In this study, the results demonstrated that the video of patient stories was powerful in sensitizing practice, challenging practice, and inspiring actions to improve dementia care. In real-life practice, relying only on decontextualized evidence and best-practice guidelines is often insufficient. As person-centered care does not provide a recipe for solving the “how to” in a given situation (Manley, McCormack, & Wilson, 2013), VRE offers a useful way to increase teams’ collective creativity, adaptive capacity, and empathetic care to make the operationalization of person-centered care possible. This supports what McCormack, Van Dulmen, Eide, Eide, and Skovdahl (2017) proposed—that person-centered care is a culture that requires the organization to embrace team engagement and reflexivity methods to enable practice development.

As Iedema et al. (2013) pointed out, practice-based evidence involves people collectively building, updating, and inventing new kinds of competence for and among themselves. In the study, the clinicians remarked that they were able to suggest creative solutions by gaining increased understanding of patients’ backgrounds, the complex clinical situation, and the relevant social context. Group reflection and sense-making with colleagues was helpful to the team, because the exercise of cointerpretation made their implicit knowledge more explicit. Private ideas could be moved out of hiding and into the open and explicitly shared space to cocreate practical and sharable knowledge. For many team members, articulating a private experience was critical for making sense of what had been happening and what might be possible in the future.

Traditional hospital systems that focus on fixing problems can keep people in a perpetual and reactive mode of firefighting (Hung et al., 2018). Often, practitioners are busy dealing with everyday tasks, which undermines their time and energy to build a positive and proactive culture of learning. VRE enables patients and staff to identify challenges and opportunities proactively for change (Iedema et al., 2013), and reflexive group sessions provide the platform for open dialogue among team members. As McCormack et al. (2017) stressed, social bonding, relationships, team dialogues, and shared visions are important for developing a person-centered care culture. Collective creativity can be learned, practiced, developed, and cultivated through regular group reflections. If done well, a cultivated environment of safe learning can continuously grow and enlarge the capacity of team members and their sense of efficacy. When the team feels confident to experiment with actions aimed at developing person-centered care, they become more resilient, effective, and productive.

Future research should further explore the benefits and challenges of filming staff care interactions with patients with dementia. We believe that much experiential knowledge and practice wisdom can be gained if staff participants have opportunities to review footage of their own practice, of how they worked together and interacted with each other in challenging clinical situations. For example, it would be useful to routinely film and play back footage of challenging events, such as those involving patient-to-staff aggression.

## Conclusion

The findings of this study offer useful insights into the dynamic process of how staff engaged in cointerpretation and colearning in video-reflexive groups. It is evident that trust and relationships were the foundation for co-inquiry and collective sense-making work. The video of patient stories filmed in situ proved to be incredibly powerful in motivating and enabling staff to improve dementia care by putting abstract person-centered care into concrete action. Staff participants in the study described the video-reflexive groups as bringing a fresh and practical approach to practice development in the hospital setting. The video clips of patient stories showed the rich complexities of everyday care and alternative possibilities to enact person-centered care. An important strength of the study is that it provides a rich description of the staff experiences in video-reflexive groups and demonstrates the value of video reflexivity in creating team commitment and strengthening collective capacities for practice development.

## References

[bibr1-2333393618785095] Adams M. Robert G. Maben J. (2015). Exploring the legacies of filmed patient narratives: The interpretation and appropriation of patient films by health care staff. Qualitative Health Research, 25, 1241–1250. 25576480 10.1177/1049732314566329 PMC4535314

[bibr2-2333393618785095] Brooker D. (2006). Person-centred dementia care: Making services better. London: Jessica Kingsley. 10.7748/nop.19.5.22.s21 27726617

[bibr3-2333393618785095] Bushe G. R. Marshak R. J. (2018). Valuing both the journey and the destination in organization development. In Jamieson D. Church A. Vogelsang J. (Eds.), Enacting values-based change: Organization development in action (pp. 87–97). New York: Palgrave Macmillan.

[bibr4-2333393618785095] Carroll K. Iedema R. Kerridge R. (2008). Reshaping ICU ward round practices using video-reflexive ethnography. Qualitative Health Research, 18, 380–390. doi:10.1177/1049732307313430 18235161

[bibr5-2333393618785095] Collier A. Sorensen R. Iedema R. (2016). Patients’ and families’ perspectives of patient safety at the end of life: A video-reflexive ethnography study. International Journal for Quality in Health Care, 28, 66–73. doi:10.1093/intqhc/mzv095 26668105

[bibr6-2333393618785095] Collier A. Wyer M. (2016). Researching reflexively with patients and families: Two studies using video-reflexive ethnography to collaborate with patients and families in patient safety research. Qualitative Health Research, 26(7), 979–993. doi:10.1177/1049732315618937 26658233

[bibr7-2333393618785095] Dewing J. (2007). Participatory research: A method of process consent with persons who have dementia. Dementia, 6, 11–25.

[bibr8-2333393618785095] Dewing J. Dijk S. (2016). What is the current state of care for older people with dementia in general hospitals? A literature review. Dementia, 15, 106–124. doi:10.1177/1471301213520172 24459188

[bibr9-2333393618785095] Dewing J. McCormack B. (2017). Tell me, how do you define person-centredness? Journal of Clinical Nursing, 26, 2509–2510. 27960045 10.1111/jocn.13681

[bibr10-2333393618785095] Francis R. (2013). Report of the Mid-Staffordshire NHS Foundation Trust Public Inquiry Volume 2: Analysis of evidence and lessons. Retrieved from https://assets.publishing.service.gov.uk/government/uploads/system/uploads/attachment_data/file/279118/0898_ii.pdf

[bibr11-2333393618785095] Gove D. Diaz-Ponce A. Georges J. Moniz-Cook E. Mountain G. Chattat R. . . . European Working Group of People With Dementia. (2018). Alzheimer Europe’s position on involving people with dementia in research through PPI (patient and public involvement). Aging & Mental Health, 22, 723–729. 28513210 10.1080/13607863.2017.1317334

[bibr12-2333393618785095] Hung L. (2015). Exploring the co-construction of meaning and power relations in walk-along interviews with individuals with dementia. The Gerontologist, 55(Suppl. 2), 23. doi:10.1093/geront/gnv161.02

[bibr13-2333393618785095] Hung L. (2017). Co-creating person-centered care in acute care (Doctoral thesis, University of British Columbia, Vancouver). Retrieved from https://open.library.ubc.ca/cIRcle/collections/ubctheses/24/items/1.0357188

[bibr14-2333393618785095] Hung L. Lee P. A. Au-Yeung A. T. Kucherova I. Harrigan M. (2016). Adopting a clinical assessment framework in older adult mental health. Journal of Psychosocial Nursing and Mental Health Services, 54(7), 26–31. 10.3928/02793695-20160616-05 27362382

[bibr15-2333393618785095] Hung L. Phinney A. Chaudhury H. Rodney P. Tabamo J. Bohl D. (2017). “Little things matter!” Exploring the perspectives of patients with dementia about the hospital environment. International Journal of Older People Nursing, 12(3), e12153. doi:10.1111/opn.12153 PMC5574000 28418180

[bibr16-2333393618785095] Hung L. Phinney A. Chaudhury H. Rodney P. Tabamo J. Bohl D. (2018). Appreciative inquiry: Bridging research and practice in a hospital setting. International Journal of Qualitative Methods, 17(1), 1–10. doi:10.1177/1609406918769444

[bibr17-2333393618785095] Iedema R. (2011). Creating safety by strengthening clinicians’ capacity for reflexivity. BMJ Quality & Safety, 20(Suppl. 1), i83–i86. 10.1136/bmjqs.2010.046714 PMC3272909 21450780

[bibr18-2333393618785095] Iedema R. (2009). New approaches to researching patient safety. Social Science & Medicine, 69(12), 1701–1704. 19853984 10.1016/j.socscimed.2009.09.050

[bibr19-2333393618785095] Iedema R. Hor S.-Y. Wyer M. Gilbert G. L. Jorm C. Hooker C. O’Sullivan M. V. N. (2015). An innovative approach to strengthening health professionals’ infection control and limiting hospital-acquired infection: Video-reflexive ethnography. BMJ Innovations, 1(4), 157–162. doi:10.1136/bmjinnov-2014-000032

[bibr20-2333393618785095] Iedema R. Mesman J. Carroll K. (2013). Visualising healthcare practice improvement: Innovation from within. London: Radcliffe.

[bibr21-2333393618785095] Kim S. K. Park M. (2017). Effectiveness of person-centered care on people with dementia: A systematic review and meta-analysis. Clinical Interventions in Aging, 12, 381–397. 28255234 10.2147/CIA.S117637 PMC5322939

[bibr22-2333393618785095] Kitwood T. (1997). Dementia reconsidered: The person comes first. Buckingham, UK: Open University Press.

[bibr23-2333393618785095] Knoblauch H. Tuma R. Schnettler B. (2015). Videography. Frankfurt: Peter Lang.

[bibr24-2333393618785095] Manley K. McCormack B. Wilson V. (2013). International practice development in nursing and healthcare. Oxford, UK: John Wiley.

[bibr25-2333393618785095] McCormack B. McCance T. (2017). Person-centred practice in nursing and health care, theory and practice. Oxford, UK: Blackwell.

[bibr26-2333393618785095] McCormack B. Van Dulmen S. Eide H. Eide T. Skovdahl K.-I. (2017). Person-centred healthcare research. Oxford, UK: John Wiley.

[bibr27-2333393618785095] Mesman J. (2011). Resources of strength: An exnovation of hidden competences to preserve patient safety. In Rowley E. Waring J. (Eds.), A socio-cultural perspective on patient safety surrey (pp. 71–92). Surrey, UK: Ashgate.

[bibr28-2333393618785095] Pink S. (2013). Doing visual ethnography. Thousand Oaks, CA: Sage.

[bibr29-2333393618785095] Reed J. (2008). Appreciative inquiry: Research for change. Thousand Oaks, CA: Sage

[bibr30-2333393618785095] Puurveen G. Phinney A. Cox S. Purvest B. (2015). Ethical issues in the use of video observations with people with advanced dementia and their caregivers in nursing home environments. Visual Methodologies, 3(2), 16–26.

[bibr31-2333393618785095] Scerri A. Innes A. Scerri C. (2016). Using appreciative inquiry to implement person-centred dementia care in hospital wards. Dementia. Advance online publication. doi:10.1177/1471301216663953 27758956

[bibr32-2333393618785095] Swaffer K. (2014). Dementia: Stigma, language, and dementia-friendly. Dementia, 13, 709–716. doi:10.1177/1471301214548143 25326220

[bibr33-2333393618785095] Venturato L. Moyle W. Steel A. (2011). Exploring the gap between rhetoric and reality in dementia care in Australia: Could practice documents help bridge the great divide? Dementia, 12, 251–267. doi:10.1177/1471301211421837 24336772

[bibr34-2333393618785095] Wyer M. Iedema R. Hor S. Y. Jorm C. Hooker C. Gilbert G. L. (2017). Patient involvement can affect clinicians’ perspectives and practices of infection prevention and control: A “post-qualitative” study using video-reflexive ethnography. International Journal of Qualitative Methods, 16(1). doi:10.1177/1609406917690171

[bibr35-2333393618785095] Wyer M. Jackson D. Iedema R. Hor S.-Y. Gilbert G. L. Jorm C.. . . Carroll K. (2015). Involving patients in understanding hospital infection control using visual methods. Journal of Clinical Nursing, 24(11-12), 1718–1729. doi:10.1111/jocn.12779 25662176

